# Mitochondrial OPA1 Deficiency Is Associated to Reversible Defects in Spatial Memory Related to Adult Neurogenesis in Mice

**DOI:** 10.1523/ENEURO.0073-23.2023

**Published:** 2023-11-08

**Authors:** Trinovita Andraini, Lionel Moulédous, Petnoi Petsophonsakul, Cédrick Florian, Sébastien Gauzin, Marlène Botella-Daloyau, Macarena Arrázola, Kamela Nikolla, Adam Philip, Alice Leydier, Manon Marque, Laetitia Arnauné-Pelloquin, Pascale Belenguer, Claire Rampon, Marie-Christine Miquel

**Affiliations:** 1 Centre de Recherches sur la Cognition Animale (CRCA), Centre de Biologie Intégrative (CBI), Université de Toulouse, Centre National de la Recherche Scientifique, Université Toulouse 3, 31400, Toulouse, France; 2 Department of Medical Physiology and Biophysics, Faculty of Medicine, Universitas Indonesia, Jakarta 10430, Indonesia

**Keywords:** adult neurogenesis, dominant optic atrophy, hippocampus, mitochondria, pattern separation, physical exercise

## Abstract

Mitochondria are integrative hubs central to cellular adaptive pathways. Such pathways are critical in highly differentiated postmitotic neurons, the plasticity of which sustains brain function. Consequently, defects in mitochondria and in their dynamics appear instrumental in neurodegenerative diseases and may also participate in cognitive impairments. To directly test this hypothesis, we analyzed cognitive performances in a mouse mitochondria-based disease model, because of haploinsufficiency in the mitochondrial optic atrophy type 1 (OPA1) protein involved in mitochondrial dynamics. In males, we evaluated adult hippocampal neurogenesis parameters using immunohistochemistry. We performed a battery of tests to assess basal behavioral characteristics and cognitive performances, and tested putative treatments. While in dominant optic atrophy (DOA) mouse models, the known main symptoms are late onset visual deficits, we discovered early impairments in hippocampus-dependent spatial memory attributable to defects in adult neurogenesis. Moreover, less connected adult-born hippocampal neurons showed a decrease in mitochondrial content. Remarkably, voluntary exercise or pharmacological treatment targeting mitochondrial dynamics restored spatial memory in DOA mice. Altogether, our study identifies a crucial role for OPA1-dependent mitochondrial functions in adult neurogenesis, and thus in hippocampal-dependent cognitive functions. More generally, our findings show that adult neurogenesis is highly sensitive to mild mitochondrial defects, generating impairments in spatial memory that can be detected at an early stage and counterbalanced by physical exercise and pharmacological targeting of mitochondrial dynamics. Thus, amplification of mitochondrial function at an early stage appears beneficial for late-onset neurodegenerative diseases.

## Significance Statement

The adult hippocampus continues to produce new neurons in mammals. These new neurons are highly sensitive to mitochondrial perturbation. Dominant optic atrophy (DOA) is a rare disease mainly caused by mutations in the gene coding the mitochondrial protein optic atrophy type 1 (OPA1). Using a mouse model of OPA1 deficiency, we found that hippocampal new neurons have dendritic spine density defects and altered mitochondrial content. We further detected impairments in spatial memory capacities relying on adult-neurogenesis. We report that these memory impairments can be corrected by physical exercise and pharmacological treatment targeting mitochondria in mice. Our results indicate that early detection of spatial memory deficits related to adult neurogenesis may allow a precocious action in pathologies involving mitochondria, such as DOA or neurodegenerative diseases.

## Introduction

Mitochondria are a dynamic population of organelles that migrate, fuse and divide. In doing so, mitochondria adapt to environmental changes at all life stages (Sharma et al., 2019; Rossmann et al., 2021). As integrative hubs, mitochondria play a particularly important role in highly plastic and fast-responding cells like neurons. The compartmentalization of neurons and their arborization, including spine-endowed dendrites, require not only active and healthy mitochondria, but also tightly regulated mitochondrial transport and dynamics, a balanced events of fusion and fission of their membranes (Flippo and Strack, 2017). Consequently, mitochondrial functions and dynamics exert a pleiotropic influence on neuronal health, from neuronal development to neurodegeneration (Khacho and Slack, 2018; Coelho et al., 2022).

Expectedly, defects in proteins critical for mitochondrial dynamics are directly associated with specific neurodegenerative diseases (Panchal and Tiwari, 2019). Among them, dominant optic atrophy (DOA), a rare disease predominantly affecting the retinal ganglion cells (RGCs) of the optic nerve, is mainly caused by mutations in the gene coding the mitochondrial protein optic atrophy type 1 (OPA1), involved in mitochondrial inner membrane fusion (Delettre et al., 2000). While homozygosity is lethal, haploinsufficiency is the main mechanism in heterozygous mutants, leading to a reduction in the OPA1 protein levels to ∼50% (Millet et al., 2016; Lenaers et al., 2021). In 20% of DOA patients, *OPA1* mutations affect not only RGCs, but also nervous tissues, leading to various neuronal extraocular symptoms (Lenaers et al., 2021).

Investigations on the role of OPA1 in neuronal functions previously demonstrated its critical involvement in neuronal maturation and plasticity (Li et al., 2004; Bertholet et al., 2013). Down-regulation of OPA1 in primary neurons indeed leads to the formation of small, fragmented mitochondria that are unevenly distributed in the dendrites and in which respiration is affected. These disturbances generate restrictions on dendritic growth, synaptogenesis and a reduction of synaptic proteins (Bertholet et al., 2013). Interestingly, although OPA1 knock-out is embryonically lethal, mouse models carrying heterozygous *Opa1* mutations exhibit late onset RGCs dysfunctions but reach adulthood without reported neuronal developmental problems (Lenaers et al., 2021). Mitochondrial dysfunctions because of OPA1 haploinsufficiency progressively build up, mainly as a pro-oxidative stress, sensitizing neurons to further challenges or insults (Millet et al., 2016; Iannielli et al., 2019; Gilkerson et al., 2021).

The role of OPA1 in neuronal maturation, prompted us to explore its influence on the maturation of neurons born in the adult hippocampus. These neurons are indeed submitted to a major challenge to properly migrate and establish synaptic connections to efficiently integrate the existing neuronal network. We hypothesized that, in a mouse model of OPA1 haploinsufficiency (Alavi et al., 2007), dysfunctional mitochondrial dynamics would affect adult hippocampal neurogenesis, leading to impairment of spatial memory processes that specifically rely on adult-born hippocampal neurons, long before any other symptom. We also evaluated whether such cognitive alterations could be corrected by enhancing brain plasticity via mitochondrial targeting.

## Materials and Methods

### Animals

We produced *Opa1*329-355del (*Opa1*+/−) mice (from Alavi et al., 2007; kind gift from Dr. B. Wissinger, University of Tübingen) in our animal facility. Genotyping was done by PCR analysis of the presence of the exon 10 of the *Opa1* gene, as previously described (Alavi et al., 2007). Splice site mutation in the *Opa1* gene of *Opa1*+/− mice leads to skipping of exon 10 and consequently, *Opa1*+/− mice show a 50% reduction in OPA1 protein levels compared with *Opa1*+/+ mice (Alavi et al., 2007; Millet et al., 2016). Males of four or eight to nine months of age were used for the anatomic and behavioral studies, respectively, to compare with previous results.

### Housing conditions

In standard conditions, mice were maintained by four to five animals per standard cage, under a 12/12 h light/dark cycle, with free access to food and water. In running conditions, mice were housed in larger cages (425 × 266 × 185 mm) containing running wheels (two wheels/four mice) to which animals had free access during three weeks.

### Ethics statement

All experiments were performed in strict accordance with the policies of the European Union (2010/63/EU) for the care and use of laboratory animals. Our animal facility is fully accredited by the national Direction of Veterinary Services (D 31-555-11, September 19, 2016) and experimental procedures conducted in this study were authorized by local ethical committees and the national Ministry for Research (#12342-2017082111489451 v6). Males of four or eight to nine months of age were used for the anatomic and behavioral studies, respectively. All efforts were made to improve animal welfare and minimize the number of animals and their suffering.

### Histologic experiments

#### Stereotactic retroviral injections

We used MML-retroviral vectors specifically transducing proliferating cells and previously described (Roybon et al., 2009); R-GFP (pCMMP-IRES2-eGFP-WPRE) for cytoplasmic expression of eGFP (kind gift of L. Roybon, Lund University) and R-mitoDsRed (pCMMP-IRES2-mitochondrial Discosoma Red-WPRE) for expression of mitochondrial matrix-targeted DsRed (Steib et al., 2014; kind gift of D. C. Lie, Helmholtz Zentrum München), at final titers of 0.7–1 × 10E9 TU/ml. Mice were anesthetized with 4% isoflurane and placed in a stereotaxic apparatus (Stoelting) with a mask connected to maintain the anesthetic with 2.5–3% isoflurane during the entire surgery. Each mouse was bilaterally injected with 1 μl of a 1:1 retrovirus mix (0.1 ml/min), into the dentate gyrus (DG; relative to bregma: anteroposterior −2 mm; lateral ±1.6 mm; ventral −2.5 mm). After injections, lidocaine was applied on the flesh before suturing the skin and the mice were placed under a heating lamp until recovery, then returned to home cage. Mice were killed three weeks after injection of virus solutions, to evaluate the morphology and mitochondrial content of 21-d-old new granule neurons after they have begun their synaptic integration (Vivar et al., 2012).

#### BrdU and Mdivi-1 administration

Mice received three intraperitoneal injections (100 mg/kg) of 5′-bromo-2 deoxyuridine (BrdU; Sigma) dissolved in 0.9% NaCl, pH 7.4, at 4-h intervals and were killed after 28 d. Mitochondrial division inhibitor 1 (Mdivi-1), 3-(2,4-dichloro-5-methoxyphenyl)−2-sulfanyl-4(3H)-quinazolinone (BML-CM127-0050; Enzo Life Sciences) was injected at 20 mg/kg [in phosphate buffer (PB)-10% dimethyl sulfoxide (DMSO)] intraperitoneally, three times a week for three weeks.

#### Tissue processing and immunohistochemistry

Mice were deeply anesthetized with do lethal solution (2 g/kg; 28 d after BrdU injection, or 21 d after viral injection), and transcardially perfused with 0.1 m PB (0.1 m) followed by 4% paraformaldehyde (PFA). Brains were removed and fixed overnight in 4% PFA, rinsed 24 h in PBS and cryoprotected in a 30% sucrose solution containing 0.1% sodium azide, at 4°C for at least 2 d. Brains were cut into 40-μm-thick coronal sections using a sliding microtome (Leica SM2010R) equipped with a freezing-stage (Physitemp BFS-3MP). Sections were kept in cryoprotectant solution at −20°C until use.

##### BrdU, NeuN, Ki67, and DCX immunohistochemistry

Free-floating brain sections were rinsed overnight in 0.1 m PBS with 0.25% Triton X-100 (PBST) before being submitted to immunohistochemistry directed against Ki67 (an endogenous marker of proliferating cells), doublecortine (DCX; a marker of immature neurons) or BrdU (marker birth-dating dividing cells). All steps were run under gentle agitation at room temperature, with PBST rinses (2 × 20 min) after each incubation. After rinses, sections were processed for quenching of endogenous peroxidases with 10% H_2_O_2_ in 10% methanol in PBS for 15 min. For BrdU staining, sections were incubated in 2 N HCl for 50 min to denature DNA followed by neutralization in 0.1 m borate buffer (pH 8.5). For all staining, nonspecific labeling was prevented by incubation in 5% normal goat serum (NGS) in PBST for 1 h. Sections were then incubated overnight in either one of the following primary antibody solutions: PBST-5% NGS with rat anti-BrdU (1:400; Biorad OBT-0030, Harlan Seralab) or PBST containing 5% NGS, 1% BSA and 0.5% Tween 20 with rabbit anti-human Ki67 (1:500; SP6 Mab, Spring Bioscience Corp, CRM325B Eurobio), or goat anti-DCX (1:100; S271390, Santa Cruz Biotechnology), or mouse anti-NeuN antibody (1:1000; MAB377, Millipore). The next day, sections were incubated for 90 min at room temperature in one of the following biotinylated secondary antibody solutions: goat anti-rat (1:400; BA9400), goat anti-rabbit (1:500; BA1000), or rabbit anti-goat (1:500; BA5000; all from Vector Laboratories) in PBST. All sections were then incubated for 90 min in avidin-biotin-peroxidase complex (1:400; Vector Laboratories Elite kit) in PBST. Staining was visualized with 0.05 m Tris-HCl buffer, pH 7.6, containing 0.025% di-amino-benzidine (DAB), 0.003% H_2_O_2_ and 0.06% nickel ammonium sulfate. Reaction was stopped by extensive rinsing with PBST containing 0.1% sodium azide (PBST-Az). Sections were mounted onto slides, counterstained with Nuclear Fast Red (Vector Laboratories), dehydrated through graded alcohols, and cover-slipped.

#### GFP immunofluorescence staining

After extensive rinsing in PBST, free-floating brain sections from retro-virus injected mice were incubated in a solution of rabbit anti-GFP (1:500; TP401, Torey Pines Biolabs) diluted in PBST overnight. Then sections were rinsed in PBST and incubated for 120 min in a solution of Alexa Fluor-488-conjugated donkey anti-rabbit (1:500; A21206, Life Technologies) in PBST. Sections were rinsed in PBST, mounted in Mowiol containing Hoechst (1:10,000; Life Technologies) and cover-slipped.

#### Cellular stereological counting

Stereological quantification of BrdU, NeuN, Ki67, or DCX -labeled (BrdU+, Ki67+, or DCX+) cells was conducted bilaterally from a 1-in-6 series of sections (240-μm interval) for BrdU staining or from a 1-in-6 series of sections (480-μm interval) for Ki67 or DCX staining, through the rostro-caudal extent of the dorsal hippocampus. Immunolabeled cells were counted manually at 40× magnification using a microscope (Leica DM6000 B) equipped with digital camera (ProgRes CFCool, Jenoptik). DCX+ immature neurons were defined as presenting processes. The experimenter was blind to the experimental groups. The density of labeled cells was calculated by dividing the number of BrdU+, Ki67+, or DCX+ cells by the granule cell layer/subgranular zone sectional volume measured with the Mercator morphometric system (Explora Nova). The total numbers of labeled cells per dentate gyrus (DG) were obtained by multiplying these densities by the reference volume, as previously described (Trouche et al., 2009). The analysis was performed on both DG in each brain, and the results represent the mean value per DG. Quantification of NeuN-labeled cells among BrdU-labeled cells in the DG was performed on a series of BrdU-labeled sections that were processed for NeuN immunostaining. NeuN-immunolabeled cells were counted manually at 40× magnification using a microscope (Leica DM6000 B) equipped with digital camera (ProgRes CFCool, Jenoptik). Quantification was limited to a total number of 50 BrdU+ cells per animal that were classified in BrdU+/NeuN+ and BrdU+/NeuN− categories.

#### Confocal analysis of dendritic spines and mitochondrial content

GFP+ neurons expressing MitoDsRed in the dorsal hippocampus were imaged with a confocal microscope. Images were deconvoluted (Huygens Essential deconvolution; Extended Data Fig. 1*A*) and analyzed using the 3D Imaris XT software (Bitplane AG; Extended Data Fig. 1*B*). Dendritic spine density and morphology were analyzed using GFP staining. Mitochondrial parameters were determined for 100 μm^3^ of GFP volume.

**Figure 1.**
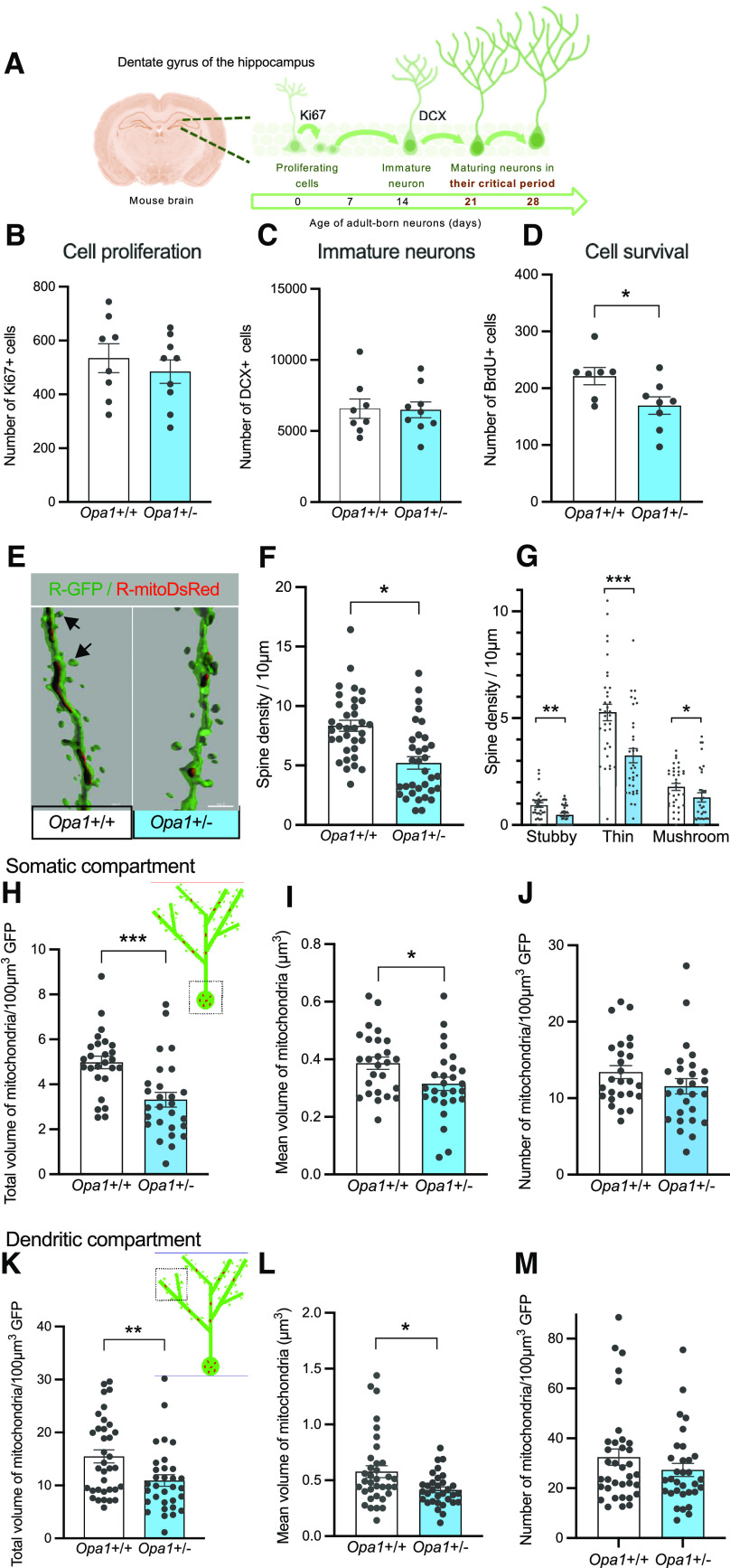
*Opa1*+/− mice show adult hippocampal neurogenesis impairments. ***A***, Schematic of the proliferation and maturation steps of adult-born neurons (in days) within the dentate gyrus of the hippocampus in mouse, and specifically their critical period (21–28 d). ***B***, Labeling against Ki67 and (***C***) DCX allowed to evaluate the number of proliferating cells and immature neurons, respectively, for each group of mice (mean values per DG, Ki67+ cells in *Opa1*+/+: 534.8 ± 53.53; in *Opa1*+/−: 484.7 ± 43.57; DCX+ cells in *Opa1*+/+: 6575 ± 686.4; in *Opa1*+/−: 6488 ± 558.4; 7–9 mice/group). ***D***, Numbers of 28-d-old BrdU+ cells (mean values per DG, *Opa1*+/+: 221.3 ± 15.09; *Opa1*+/−: 169.5 ± 15.37; 7–9 mice/group, *p = 0.0329; unpaired *t* test). ***E***, Representative 3D reconstruction images showing GFP+ (green) dendritic spines (arrows) and mitoDsRed+ (red) mitochondria. ***F***, Density of GFP+ dendritic spines (*Opa1*+/+: 8.34 ± 0.46/10 μm dendrite; *Opa1*+/−: 5.22 ± 0.51/10 μm dendrite) and (***G***) spine subtypes (stubby: *Opa1*+/+: 0.93 ± 0.10 vs *Opa*1+/−: 0.47 ± 0.07 spines/10 μm; thin: *Opa1*+/+: 5.27 ± 0.3 vs *Opa1*+/−: 3.25 ± 0.3 spines/10 μm; mushroom: *Opa1*+/+: 1.78 ± 0.16 vs *Opa1*+/−: 1.29 ± 0.2 spines/10 μm; *p < 0.05, **p < 0.01, ***p < 0.001; two-tailed Mann–Whitney test). ***H***, ***M***, Mitochondrial parameters in the somatic and dendritic compartments of 21-d-old neurons. ***H***, ***K***, Total mitochondrial biomass per somatic or dendritic GFP+ volume. Somas: ***G***, *Opa1*+/+: 4.98 ± 0.27; *Opa1*+/−: 3.32 ± 0.33; Dendrites: ***J***, *Opa1*+/+: 15.49 ± 1.23; *Opa1*+/−: 10.93 ± 1.08. ***I***, ***L***, Mean volume of individual somatic or dendritic mitochondria. Somas: ***H***, *Opa1*+/+: 0.39 ± 0.02 μm^3^; *Opa1*+/−: 0.32 ± 0.02 μm^3^; Dendrites: ***K***, *Opa1*+/+: 0.58 ± 0.05 μm^3^; *Opa1*+/−: 0.41 ± 0.02 μm^3^. ***K***, ***M***, Number of mitochondria per somatic or dendritic GFP+ volume. Somas: ***I***, *Opa1*+/+: 13.41 ± 0.85 particles/100 μm^3^; *Opa1*+/−: 11.57 ± 1.01 particles/100 μm^3^; Dendrites: ***L***, *Opa1*+/+: 32.47 ± 3.28 particles/100 μm^3^; *Opa1*+/−: 27.35 ± 2.64 particles/100 μm^3^. Mean ± SEM; five mice/group, five to seven somas/mouse, six to eight dendritic segments/mouse (*p <* *0.05, **p <* *0.01, ***p <* *0.001; two-tailed Mann–Whitney test). See Extended Data Figure 1-1, mitochondria in GFP+ adult born neurons from mouse dentate gyrus.

10.1523/ENEURO.0073-23.2023.f1-1 Extended Data Figure 1-1 Mitochondria in GFP+ adult born neurons from mouse dentate gyrus. Images of MitoDsRed+ mitochondria inside GFP+ adult-born neurons in the dentate gyri from *Opa1*+/+ and *Opa1*+/− were captured from mouse brain sections, using a confocal microscope with a 63x oil lens and digital zoom of 6. A representative image of a 3D-projection from z-series acquired from each genotype, is shown, with inverted colors, after deconvolution using the Huygens Essential deconvolution software (SVI) (***A***) and after segmentation using the Imaris surface tool (***B***). Download Figure 1-1, EPS file.

#### Analysis of dendritic spine density and shape

Spines were analyzed within the distal dendritic part of adult hippocampal new neurons to describe the morphologic synaptic integration of these neurons. The distal dendritic compartment was defined as the dendritic shaft located between the beginning of the middle molecular layer and the end of the outer molecular layer, comprising mainly segments located after the second dendritic branching point. Spine analysis included spine density (number of spines/10-μm dendritic length) and morphologic classification. The 3D reconstruction of the dendrites was performed with the Imaris software. Spines volume and number were determined with the Imaris surface tool module, while dendritic length was measured with the Imaris Filament tracer module. Dendritic spines were defined as protrusions from the dendritic shaft and were categorized based on their individual volume and morphology into three types, excluding filopodia: thin (protrusion with a neck and a head <0.1 μm^3^), stubby (protrusion with no obvious neck or head) or mushroom (protrusion with a neck and a head >0.1 μm^3^; Harris et al., 1992). Spine density was calculated by dividing the total number of spines by the length of the dendritic segment. Confocal imaging and data analysis were performed blindly to experimental conditions.

#### Mitochondrial content of adult-born hippocampal neurons

MitoDsRed-positive mitochondria inside GFP-positive neurons were analyzed in the somatic and distal (as above defined) dendritic compartments. The total volume of mitochondria, their mean volume and their number were automatically determined using the Imaris software (Bitplane). For both the somatic and distal dendritic compartments, mitochondrial total volume was normalized for 100 μm^3^ of GFP volume of the corresponding compartment, mitochondrial number and mitochondrial mean volumes were calculated within the same portions. Briefly, GFP volume, reflecting the total volume of the soma or dendritic shaft, was determined using the Imaris surface tool with the following parameters: smoothing enabled (surface detail 0.0687), absolute intensity thresholding, filtering by number of voxel (>10). Mitochondria were then segmented only within the GFP volume using the Imaris surface tool with the following parameters: smoothing enabled (surface detail 0.1), background subtraction thresholding (largest sphere diameter = 0.1 μm), filtering by volume (>0.01) and intensity (>10). Confocal imaging and data analysis were performed blindly to the experimental condition.

### Behavioral experiments

Behavioral tests were performed by experimenters blind to genotypes, using published protocols. *Opa1+/*+ and *Opa1*+/− mice were tested for anxiety-related and exploratory behaviors. The nonspatial (cue) version of the Barnes maze (vision-guided) and the object recognition task allowed to evaluate hippocampal-independent memory. The spatial version of the Barnes maze, the pattern separation and object location tasks were used to evaluate hippocampal-dependent memory. Consequences of voluntary exercise were assessed using a standard running procedure. All parameters were measured using Ethovision software (Noldus).

### Detailed protocols

A comprehensive battery of tests was deployed to evaluate several aspects of mice behavior. Before each experimental session, mice received 3–4 d of handling to get habituated to the experimenter. Mice were pseudo-randomly distributed into batches of 8–14 mice with counterbalanced numbers of *Opa1*+/+ and *Opa1*+/− genotypes, experimenters being blind to genotypes. All experiments were videorecorded and locomotor activity was automatically analyzed using Ethovision XT software (Noldus) except tasks involving objects, for which the exploration time was recorded manually. The setups were cleaned thoroughly with 70% ethanol followed by water between each mouse session, to ensure the absence of olfactory cues.

#### General behavior

Anxiety-related behavior was assessed using an elevated plus maze. Locomotor activity was recorded during the 10-min familiarization phase of the object location or recognition tasks, using an empty circular arena (40 cm in diameter) devoid of visual cues.

#### Spatial and cued navigation in the Barnes maze

**Figure 2.**
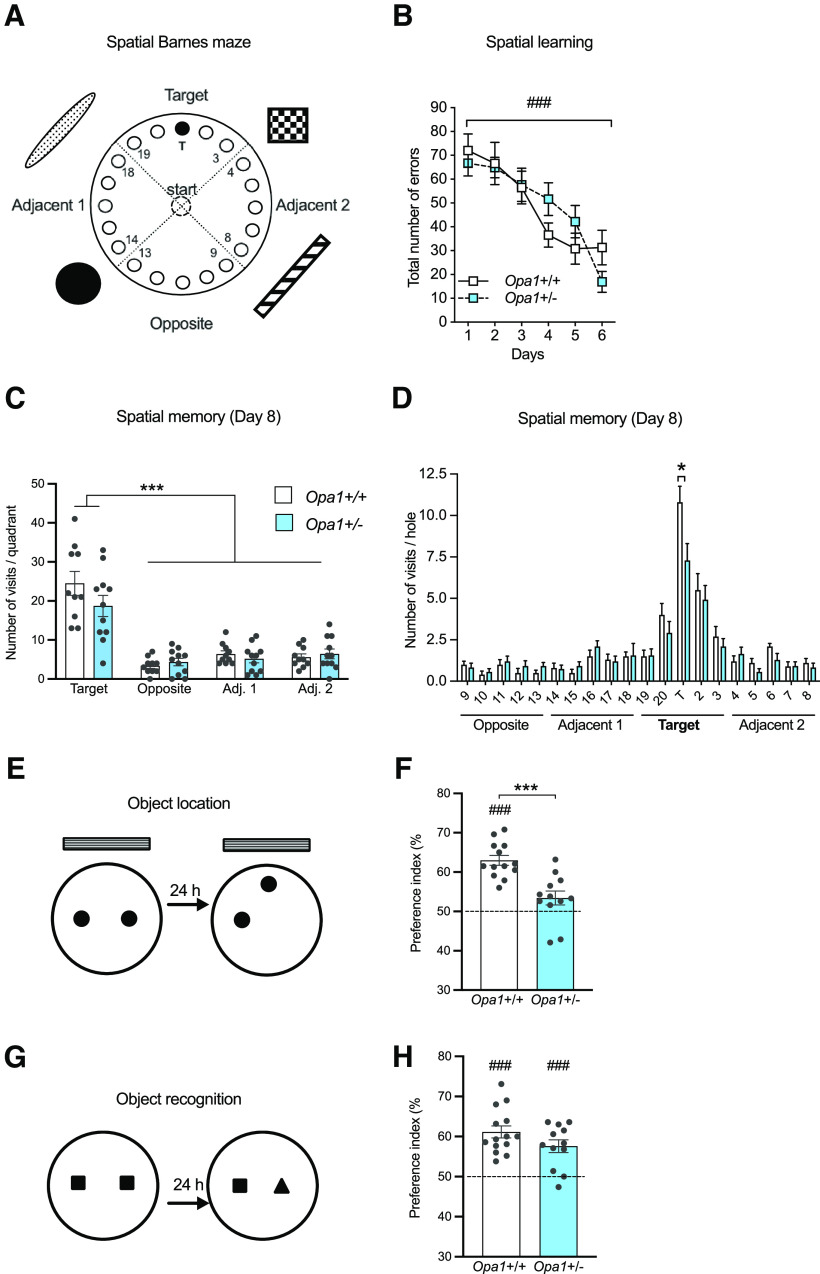
*Opa*1+/− mice have impaired spatial memory but intact nonspatial memory. ***A***, Schematic setup of the spatial version of the Barnes maze. ***B***, Number of errors before entering into the escape hole across training sessions, *Opa1*+/+ (*n *=* *10) and *Opa1*+/− (*n *=* *11), genotype, *p *=* *0.88; two-way ANOVA with repeated measures). ***C***, Two days after the last training session (day 8), memory was evaluated by a spatial memory probe test. Mice from both genotypes visited significantly more often the holes located in the target quadrant than in the other quadrants (target quadrant: ***p < 0.0001; two-way ANOVA). ***D***, *Opa1*+/− mice visited the target hole significantly less often than the *Opa1*+/+ mice (*Opa1*+/+: 10.80; *Opa1*+/−: 7.27; *p <* *0.05; unpaired *t* test). ***E***, Schematic of the object location task. ***F***, Preference for the displaced object compared with chance level: *Opa1*+/+: 62.98 ± 1.24%; *n *=* *13, ###*p *<* *0.0001; *Opa1*+/−: 53.40 ± 1.77%; *n *=* *12, p>0.05; index versus 50%, one-sample *t* test. Compared with *Opa1*+/+ mice, mutant mice exhibited a spatial memory deficit (***p <* *0.001; unpaired *t* test). ***G***, Schematic of the object recognition task. ***H***, During memory testing, both genotypes explored preferentially the novel object than the familiar one (*Opa1*+/+, 61.14 ± 1.51%, *n *=* *14; *Opa1*+/−, 57.60 ± 1.57%, *n *=* *12, ###*p *<* *0.001, respectively; index vs 50%, one-sample *t* test). In ***F*** and ***H***, dotted lines indicate equal exploration of the two objects. See Extended Data Figure 2-1, intact anxiety-related and locomotor behavior in *Opa1*+/− mice. See Extended Data Figure 2-2, *Opa1*+/− mice display intact performances in the nonspatial Barnes maze. See Extended Data Figure 2-3, long-term spatial memory is less accurate in *Opa1+/−* mice than in *Opa1+/+* mice; *Opa1*+/+ and *Opa1*+/− mice express similar interest for all objects.

This behavioral paradigm relies on the innate preference of rodents to escape bright light and open spaces (Barnes, 1979; Bach et al., 1995). The circular Barnes maze (90 cm in diameter) contains 20 holes (5 cm in diameter, 8 cm apart) evenly located around the perimeter. It is elevated 90 cm from the floor and receives ∼800 lux from a centered bright light, to motivate the mice to escape through the escape cage. The target hole is connected with a tube to an escape cage located beneath the maze. Other cages containing bedding mix from the escape cage, but not connected to any of the holes, were positioned underneath the maze to avoid olfactory bias. We used two paradigms, and independent groups of mice, to respectively evaluate hippocampal-independent nonspatial and hippocampal-dependent spatial navigation. For the nonspatial (cued, hippocampal-independent) version, a ball (10 cm in diameter) was placed near the target hole, and the position of the cued target hole varied pseudo-randomly over days (Extended Data Fig. 2-2*A*). Here, mice learnt the association between the cue (the ball) and the target hole. For the spatial version of the task (hippocampal-dependent), visual cues were positioned on the walls surrounding the maze (Fig. 2*A*). The location of the target hole remained the same across trials and days, so that the mice had to learn the location of the target hole based on the distal cues.

10.1523/ENEURO.0073-23.2023.f2-1 Extended Data Figure 2-1 Intact anxiety-related and locomotor behavior in *Opa1*+/− mice. OPA1 deficiency has no impact on anxiety-like behavior measured by (***A***) the time spent in the open arms of the elevated plus maze (in %, *Opa1*+/+: 29.01 ± 4.27; *Opa1*+/−: 30.72 ± 2.28, *t *=* *0.34, df =* *25, *p *=* *0.734; *t* test). Mice from both genotypes show similar locomotor activity in the open-field, evaluated by (***B***) the time spent in the center area (in %, *Opa1*+/+: 31.07 ± 2.03; *Opa1*+/−: 30.73 ± 2.46, *t *=* *0.11, df =* *25, *p *=* *0.915; *t* test), and (***C***) the total distance travelled (in cm, *Opa1*+/+^:^ 3246 ± 437.7; *Opa1*+/−: 3423 ± 145.1, *p* = 0.2388; *U* = 66, two-tailed Mann–Whitney test). *Opa1*+/+, *n *=* *14; *Opa1*+/−, *n *=* *13. Download Figure 2-1, EPS file.

10.1523/ENEURO.0073-23.2023.f2-2 Extended Data Figure 2-2 *Opa1*+/− mice display intact performances in the non-spatial Barnes maze. ***A***, Schematic setup of the non-spatial (cued) Barnes maze. ***B***, During the five days of training, the number of errors before entering the escape hole decreased across training sessions for mice of both genotypes, indicating they learned to locate the escape hole (*session/day*: *F*_(4,88)_ = 17.82, ###*p *<* *0.0001; two-way ANOVA with repeated measures). No difference was found between genotypes (*F*_(1,22)_ = 0.002, *p *=* *0.96; two-way ANOVA with repeated measures). ***C***, During the non-spatial probe test held 24 h after training (Day 6), both groups of mice visited more often the holes located in the target quadrant than in each of the other quadrants (*F*_(3,88)_ = 51.35, ***p <* *0.0001; two-way ANOVA) and no difference was observed between genotypes (*F*_(1,88)_ = 2.355, *p* = 0.128; two-way ANOVA). ***D***, *Opa1*+/+ and *Opa1*+/− mice exhibited a similar preference for the target hole (*t* = 0.4089, df =* *38, *p *=* *0.6849; unpaired *t* test). *Opa1*+/+, *n *=* *12; *Opa1*+/−, *n *=* *12. Download Figure 2-2, EPS file.

**Figure 3.**
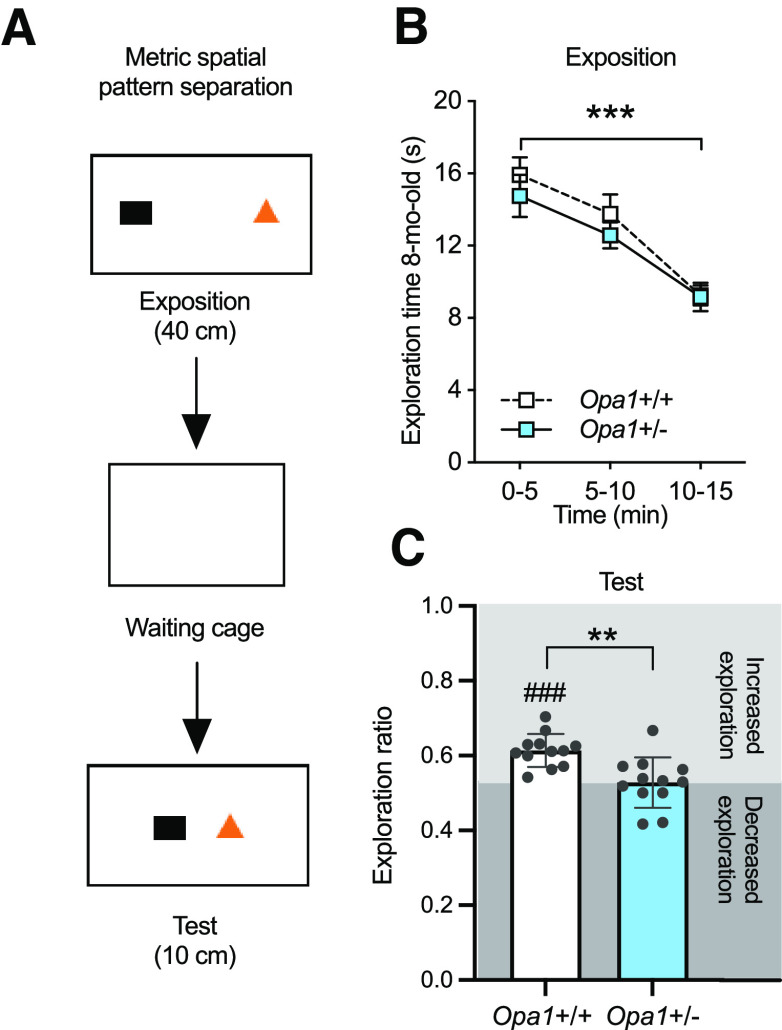
*Opa1*+/− mice show deficits in metric spatial pattern separation. ***A***, Schematic of the pattern separation procedure. ***B***, Exploration time during exposition phase (session: ***p <* *0.0001; two-way ANOVA with repeated measures). No genotype effect was observed. ***C***, Exploration ratio during memory test in *Opa1*+/+ mice (61.4 ± 1.3%, *n *=* *12, *t* = 8.914, df* *=* *11, ###*p *<* *0.0001, one-sample *t* test) and *Opa1*+/− mice (52.8 ± 1.9%, *n *=* *12, *t *=* *1.430, df* *=* *11, *p* = 0.181, one-sample *t* test). Performances in pattern separation were significantly different between genotypes (**p <* *0.01; unpaired *t* test). Dotted line indicates equal exploration of the two objects.

10.1523/ENEURO.0073-23.2023.f2-3 Extended Data Figure 2-3 Long-term spatial memory is less accurate in *Opa1*+/− mice than in *Opa1*+/+ mice. ***A***, During the spatial memory probe test held 7 d after training (Day 13), mice from both genotypes visited significantly more often the holes located in the target quadrant than those located in the other quadrants (*F*_(3,76)_ = 56.56, ***p <* *0.0001; two-way ANOVA). However, *Opa1*+/− visited significantly less often the holes of the target quadrant (*Opa1*+/+: 22.40, *Opa1*+/−, 16.45; #*p *=* *0.0482; unpaired *t* test). ***B***, This effect was strictly attributable to a difference in the number of visits to the target hole between *Opa1*+/− and *Opa1*+/+ mice (*Opa1*+/+: 9.90, *Opa1*+/−, 6.27; *t *=* *3.433, df =* *19, **p = 0.0028; unpaired *t* test), revealing a less precise spatial memory in mutant mice. *Opa1*+/+, *n *=* *10; *Opa1*+/−, *n *=* *11. *Opa1*+/+ and *Opa1*+/− mice express similar interest for all objects. Mice from both genotypes spent the same amount of time exploring the left and right objects during the acquisition phase of the object location (***C***) and object recognition (***D***) tasks. Mean exploration time was not different between genotypes, indicating that both groups of mice had a similar interest for each object. Object location: *Opa1*+/+, *n* = 13, left object: 15.08 ± 1.5, right object: 15.46 ± 1.33; *Opa1*+/−, *n *=* *12, left object: 14.92 ± 1.44, right object: 14.75 ± 2.09. Object recognition: *Opa1*+/+, *n* = 14, left object: 16.79 ± 1.80, right object: 16.79 ± 1.89; *Opa1*+/−, *n *=* *12, left object: 16.67 ± 1.50, right object: 16.83 ± 1.40. Download Figure 2-3, EPS file.

We used a modified protocol of Sunyer (Sunyer et al., 2007). Briefly, after 3 consecutive days of handling, mice were submitted to either the cued or spatial protocol and exploration was manually recorded. First, mice were familiarized to the setup and to descending into the escape cage through the tube. Then animals received four trials per day during 5 d (cued) or 6 d (spatial). For each trial, the mouse was released from the center of the maze and given 5 min to enter the target hole, after which, in case of failure, the mouse was gently guided to the target hole. For each trial, the total number of errors made before first entrance into the target hole was recorded. Errors are scored each time a mouse visits (i.e., dips its head into a hole) a hole not connected to the escape cage or the target hole without descending into the escape cage. Probe test for the cued learning was conducted 1 d after the last acquisition trial whereas mice trained in the spatial version of the test were submitted to two probe tests held 2 and 7 d after training completion (on days 8 and 13, respectively). In order to prevent memory extinction, animals were given three learning trials after the first probe test (data not shown). The target hole was disconnected from the escape cage and the mouse was given 5 min to explore the maze during which numbers of visits to each hole were counted.

#### Object location

This task evaluates spatial hippocampal-dependent memory and relies on the ability of rodents to discriminate between a novel and a familiar spatial location. Performances in this task are sensitive to the depletion of hippocampal adult neurogenesis. This test takes place in a circular arena (40 cm in diameter) containing a visual cue (rectangular striped pattern), surrounded by a white curtain, and is conducted in three phases: familiarization, exploration, and test. One day before acquisition, each mouse was familiarized to the empty arena during 10 min. The next day, two identical objects are placed in the middle of the arena 20 cm apart. The mice are allowed to explore for 10 min during which the time spent sniffing the two objects was recorded. Mice that did not reach the criterion of 25 s of cumulated exploration for both objects were excluded. As a result, one mouse of each genotype was excluded from the analysis. On the test phase held 1 d later, copies of the same objects were exposed and one of the two objects was moved to a novel location. The position (left or right) of the displaced object was randomized to reduce bias toward a particular location. Mice were allowed to explore the objects during 10 min and the preference index for the displaced object was calculated as described below.

#### Object recognition

This task evaluates hippocampal-independent memory and assesses the ability of rodents to discriminate between a familiar and a novel object (Ennaceur and Delacour, 1988; Ennaceur et al., 1997). Performances in this task are not sensitive to the depletion of hippocampal adult neurogenesis (Ennaceur et al., 1997; Goodman et al., 2010). A setup similar to the one described for the novel object location test was used, but the pattern was removed. The familiarization phase was identical to the object location task. The next day, mice were allowed to explore two identical objects placed in the center of the arena. The object exploration criterion was the same than for the object location task. Recognition memory was tested the following day. Mice were reintroduced for 10 min in the arena containing one familiar object and one novel object (left or right counterbalanced) which positions were identical to the acquisition phase. The preference for the novel object was calculated as described below.

#### Preference index

Object exploration was defined as the time spent actively sniffing or interacting with the object within a distance of 2 cm maximum. Measurement of the time spent exploring the displaced or novel object was expressed as a percentage of time spent exploring the displaced or novel object, related to the total exploration time for both objects during the test phase (in %, preference index; 50% is chance level).

#### Metric pattern separation test

This task evaluates memory processes that strongly rely on the dentate gyrus. Our protocol is based on the one described in previous study (Hunsaker et al., 2009). The setup consisted of a rectangular arena (60 × 40 × 30 cm) devoid of visual patterns. On the first day, mice were allowed to explore the empty arena for 20 min in groups of four mice, followed by 5 min of individual exploration. The next day, each mouse was allowed to explore for 15 min two different objects, placed 40 cm apart in the arena (exposition phase) during which exploration was measured by blocks of 5 min. Then, the mouse was placed in a waiting cage (standard cage with bedding material) during 20 min, and the objects were repositioned at a 10 cm distance. The mouse was then put back into the arena for a 5 min (test phase), during which it was free to re-explore the objects in their new metric configuration. Mice that did not reach the criterion of 20 s of total exploration time for both objects during the sample phase were removed from the experiment (one for each genotype in the object location task, while two *Opa1*+/− mice were excluded in the object recognition task). Mice performances were evaluated via an exploration ratio calculated as follows: (exploration during 5 min of test phase)/(exploration during 5 min of test phase + exploration during the last 5 min of exposition phase). This constrained all the values between 0 and 1. Thus, an increased exploration during test phase was reflected by a ratio >0.5, while a decreased exploration (or habituation) was reflected by a ratio <0.5. Increased exploration of the new configuration demonstrates the ability of the mice to detect metric changes in the relationships between objects, as described by Hunsaker et al. (2009).

### Statistics

Data analysis was performed with Prism software (GraphPad.9). Morphologic data analyses were evaluated with unpaired *t* test, or Mann–Whitney test whether the distribution data were not normal, to compare parameters between groups. Behavioral analyses were evaluated with one-sample *t* test to compare preference index with chance level 50% or with 0.5 ratio and unpaired *t* test to compare between genotypes. Repeated measurement two-way ANOVA with Bonferroni *post hoc* analyses were used when allowed. Mean ± SEM, appropriate analyses, parameters, and *p* values are indicated in Results. Threshold for significance was set at *p* < 0.05. See Extended Data Table 1-1.

10.1523/ENEURO.0073-23.2023.t1-1 Extended Data Table 1 Data structure, test and power are shown for each figure. Download Table 1-1, XLS file.

### Data availability

The data that support the findings of this study are available from the corresponding author, on reasonable request.

## Results

### Adult hippocampal neurogenesis is impaired in *Opa1*+/− mice

In the dorsal hippocampus of adult mice, new neurons are generated every day in the dentate gyrus, maturate and migrate to become granule cells (Fig. 1*A*). The period between three and five weeks after their birth is a critical period, because newly-born neurons display a particularly distinct function from their mature state, as they are highly excitable and likely to be recruited into neuronal networks supporting memory.

Using immunohistochemistry against specific markers of cell proliferation (Ki67) and of immature neurons (doublecortin), we found that numbers of Ki67-labeled (Ki67+; Fig. 1*B*) and doublecortin-labeled (DCX+; Fig. 1*C*) cells in the dentate gyrus were similar in *Opa1*+/+ and *Opa1*+/− mice (Ki67+ cells in *Opa1*+/+: 534.8 ± 53.53; in *Opa1*+/−: 484.7 ± 43.57; DCX+ cells in *Opa1*+/+: 6575 ± 686.4; in *Opa1*+/−: 6488 ± 558.4; eight and nine mice per group). This indicates that OPA1 deficiency has no consequence on neural stem cell proliferative activity nor on neuronal differentiation.

Further, the survival of adult-generated hippocampal cells was assessed by evaluating the absolute number of BrdU+ cells in the dentate gyrus, 28 d after BrdU injection. Interestingly, we found lower numbers of 28-d BrdU+ cells in *Opa1*+/− mutant mice compared to control mice indicating a reduced survival of adult generated cells (*Opa1*+/+: 221.3 ± 15.09; *Opa1*+/−: 169.5 ± 15.37; seven and eight mice per group, *p = 0.0329, *t* = 2.387, *df* =13, unpaired *t* test; Fig. 1*D*). Immunostaining of the neuronal marker NeuN revealed that among these BrdU+ cells, the same proportion, about 70%, had become new neurons in *Opa1*+/− and *Opa1*+/+ mice (data not shown; *Opa1*+/+: BrdU+NeuN+ 69.4%, mean NeuN-BrdU+ cells over 49 counted cells: 34 ± 2.1; *Opa1*+/−: BrdU+NeuN+ 74.3%, mean NeuN-BrdU+ cells over 49 counted cells: 36.63 ± 1.86; seven and eight mice per group). Thus, OPA1 deficiency does not seem to affect neural cell fate. Altogether, these data suggest that the hippocampus of OPA1-deficient mice contains a reduced number of adult-generated neurons.

Since the synaptic integration of adult-generated hippocampal neurons is critical for their survival and function (Vivar et al., 2012; Aimone et al., 2014), we next evaluated the dendritic spine density of adult-born neurons. Twenty-one days after GFP-retrovirus injection, we found that *Opa1*+/− genotype is associated with a significant reduction of dendritic spine density on GFP-labeled adult-born granule neurons compared with control mice (*Opa1*+/+: 8.34 ± 0.46/10 μm dendrite;*Opa1*+/−: 5.22 ± 0.51/10 μm dendrite, ***p <* *0.0001; *U *=* *246.5, two-tailed Mann–Whitney test; Fig. 1*E*,*F*). Going further, we classified these spines into four morphologic types based on their morphology: stubby, filopodia, thin and mushroom (see Materials and Methods). As opposed to the filopodia type (filopodia: *Opa1*+/+: 0.35 ± 0.06 vs *Opa1*+/−: 0.2 ± 0.05 spines/10 μm *p *=* *0.0633, *U *=* *435.5), we found that stubby, thin and mushroom types were significantly depleted on *Opa1*+/− mice GFP-labeled neurons (stubby: *Opa1*+/+: 0.93 ± 0.10 vs *Opa1*+/−: 0.47 ± 0.07 spines/10 μm; thin: *Opa1*+/+: 5.27 ± 0.3 vs *Opa1*+/−: 3.25 ± 0.3 spines/10 μm; mushroom: *Opa1*+/+: 1.78 ± 0.16 vs *Opa1*+/−: 1.29 ± 0.2 spines/10 μm; stubby, **p =* *0.0027, *U *=* *337.5; thin, ***p <* *0.0001 *U *=* *274; mushroom, *p =* *0.0356, *U *=* *407, two-tailed Mann–Whitney test; Fig. 1*G*).

In summary, we found that under OPA1 deficiency, fewer adult-born cells survive through the critical period, and the surviving adult-born neurons exhibit impaired synaptic connectivity. Because mitochondrial functions are critical for adult neurogenic processes, we next examined the mitochondrial content of adult-generated neurons in *Opa1*+/− mice.

### Maturing adult-born neurons from *Opa1*+/− mice show altered mitochondrial biomass

In this *Opa1*+/− mouse model, nothing is known on the mitochondrial content of adult-generated hippocampal neurons. In neurons, mitochondrial biogenesis occurs primarily in the soma from where mitochondria are distributed to other compartments, including dendrites. Following intrahippocampal injection of retroviruses encoding MitoDsRed or GFP, Mito-DsRed-labeled mitochondria were quantified in the somatic and dendritic compartments of 21-d-old GFP-labeled adult-born neurons (Fig. 1*E*,*H–M*; illustrated in Extended Data Fig. 1-1). In both the neuronal somatic and dendritic compartments, the total mitochondrial biomass (per 100 μm^3^ of GFP+ cell volume) was 30 to 40% lower in *Opa1*+/− compared to control mice [Somas: *Opa1*+/+: 4.98 ± 0.27; *Opa1*+/−: 3.32 ± 0.33, ***p = 0.0002, *U* = 145 (Fig. 1*H*); Dendrites: *Opa1*+/+: 15.49 ± 1.23; *Opa1*+/−: 10.93 ± 1.08; **p = 0.0079, *U* = 350 (Fig. 1*K*); two-tailed Mann–Whitney test]. However, the number of mitochondria per volume of GFP+ soma or dendrite was not significantly different in *Opa1*+/− and control mice [Somas: *Opa1*+/+: 13.41 ± 0.85 particles/100 μm^3^; *Opa1*+/−: 11.57 ± 1.01 particles/100 μm^3^, *p* = 0.148, *U* = 269 (Fig. 1*J*); Dendrites: *Opa1*+/+: 32.47 ± 3.28 particles/100 μm^3^; *Opa1*+/−: 27.35 ± 2.64 particles/100 μm^3^, *p* = 0.334, *U* = 482.5 (Fig. 1*M*), two-tailed Mann–Whitney test]. Thus, the overall decrease in total mitochondrial volume is rather attributable to the lower mean volume of individual mitochondria observed in the soma and dendrites of adult-generated neurons of *Opa1*+/− mice compared to control [Somas: *Opa1+/+:* 0.39 ± 0.02 μm^3^; *Opa1*+/−*:* 0.32 ± 0.02 μm^3^, *p = 0.03, *U* = 230, two-tailed Mann–Whitney test (Fig. 1*I*); Dendrites: *Opa1*+/+: 0.58 ± 0.05 μm^3^; *Opa1*+/: 0.41 ± 0.02 μm^3^, *p = 0.024, *U* = 381.5, two-tailed Mann–Whitney test (Fig. 1*L*)]. Thus, OPA1 deficiency results in reduced mitochondrial content in adult-born neurons during their critical developmental period.

Overall, this cellular analysis reveals impaired adult hippocampal neurogenesis in *Opa1*+/− mice. The remarkable and unique plastic properties of the new neurons during their critical period have been reported (i.e., between three and five weeks after birth), suggesting that they are ideally suited to sustain memory mechanisms (Schmidt-Hieber et al., 2004; Tashiro et al., 2007; Aasebø et al., 2011). Supporting this idea, their crucial contribution to hippocampal-dependent forms of memory has been largely documented in the literature (Sahay et al., 2011; Toda et al., 2019).

Because adult-born neurons of *Opa1*+/− mice display impaired synaptic connectivity and a reduced mitochondrial content during the critical period of their development, we hypothesized that these mice may exhibit hippocampal-dependent memory impairments by middle age, when adult neurogenesis deficits have accumulated. To address this issue, we submitted eight- to nine-month-old *Opa1* mutant mice to a comprehensive battery of behavioral tests.

### *Opa1*+/− mice have normal general behavior and vision

In order to reveal clear possible defects, mice of both genotypes were tested at middle age (eight to nine months old). At the behavioral level, *Opa1*+/− mice showed similar anxiety level and locomotor activity than control littermates (Extended Data Fig. 2-1*A–C*), consistent with a previous report (Caffin et al., 2013). Moreover, these animals displayed normal visual learning and memory in the vision-dependent nonspatial Barnes maze (Extended Data Fig. 2-2*A–D*). Similar results were obtained with four-month-old animals (data not shown). These findings allowed us to proceed with visually-guided memory tasks.

Given the altered hippocampal neurogenesis of *Opa1*+/− mice, we next focused on hippocampal-dependent, neurogenesis sensitive, behavioral tasks.

### *Opa1*+/− mice show spatial memory deficits

Next, we evaluated spatial (hippocampal-dependent) and nonspatial (hippocampal-independent) memory of OPA1-deficient mice. In the spatial version of the Barnes maze (Fig. 2*A*), both *Opa1*+/+ and *Opa1*+/− mice showed spatial learning (session: *F*_(5,95)_ = 25.92, ***p < 0.0001, two-way ANOVA with repeated measures; Fig. 2*B*) and memory capacities (target quadrant: *F*_(3,76)_ = 48.8, ^***^p <* *0.0001; two-way ANOVA; Fig. 2*C*). However, compared with control mice, *Opa1*+/− animals displayed a less precise memory (*p =* *0.022; *t* = 2.502, df =* *19, unpaired *t* test; Fig. 2*D*), confirmed at a later delay [#*p *=* *0.0482; *t *=* *2.111, df* *=* *19, unpaired *t* test (Extended Data Fig. 2-3*A*); **p =* *0.0028; *t *=* *3.433, df* *=* *19, unpaired *t* test (Extended Data Fig. 2-3*B*)]. Thus, our data indicate that *Opa1*+/− mice are capable of spatial learning and have long-term memory, but that their memory persistence or robustness is lower than that of control mice.

Then, these mice were submitted to the object location task (Fig. 2*E*), which evaluates the ability of mice to discriminate objects in a novel versus a familiar location. This task assesses spatial memory and is sensitive to adult neurogenesis depletion. In the exploration phase, *Opa1*+/− and control littermates spent a similar percentage of time exploring each object and the average total time spent exploring both objects was not different across genotypes (Extended Data Fig. 2-3*C*), indicating a similar interest for both objects. During the 24-h test phase, one of the objects was moved to a new location and spatial memory was evaluated (Fig. 2*E*). Control *Opa1*+/+ mice explored preferentially the displaced object, while *Opa1*+/− mice showed no preference for the displaced object (*Opa1*+/+, ###*p *<* *0.0001, *t *=* *10.450, df* *=* *12; *Opa1*+/−, *p *=* *0.808, *t *=* *1.922, df* *=* *11; index vs 50%, one-sample *t* test; Fig. 2*F*). These data reveal that *Opa1*+/−-deficient mice exhibit an altered spatial memory compared with control littermates (*t* = 4.489, df* = *23, ***p =* *0.0002; two-tailed unpaired *t* test; Fig. 2*F*). Altogether, these results reveal that *Opa1*+/−-deficient mice exhibit alterations in spatial memory capacities compared with control littermates when they reach middle age.

Animals were also tested in the object recognition task, which evaluates nonspatial memory and is insensitive to hippocampal neurogenesis depletion. The nonspatial object recognition task relies on the spontaneous preference of rodents for novelty and assesses the ability of mice to discriminate between a novel and a familiar object (Fig. 2*G*). Mice of both genotypes exhibited similar interest for both objects during acquisition (Extended Data Fig. 2-3*D*). Recognition memory was tested 24 h after the acquisition phase, when a new object replaced one of the familiar objects. Both groups of mice spent more time exploring the new object than the familiar one, indicating intact recognition memory (*Opa1*+/+, ###*p *<* *0.0001, *t *=* *7.394, df* *=* *13; *Opa1*+/−, ###*p *<* *0.0005, *t *=* *4.828, df* *=* *11, respectively; index vs 50%, one-sample *t* test; Fig. 2*H*). Moreover, both groups exhibited similar levels of performances indicating no genotype difference. Hence, OPA1 deficiency results in impairment of spatial memory while sparing nonspatial memory.

In order to further evaluate the impact of altered adult neurogenesis on cognitive performances of *Opa1*+/− mice, animals were then submitted to the metric spatial pattern separation task. This test evaluates the ability of mice to spontaneously detect a minor change in the distance separating two objects (Fig. 3*A*; Goodrich-Hunsaker et al., 2005; Hunsaker and Kesner, 2008) and strongly relies on the adult-born neurons of the dentate gyrus (Kesner et al., 2014). All mice displayed habituation to the objects during the exposition phase, as demonstrated by a decrease in exploration duration during the 15 min-exposition phase (session: *F*_(1.407,30.95)_ = 25.36, ***p < 0.0001; two-way ANOVA with repeated measures; Fig. 3*B*). Moreover, the total time spent exploring both objects was not different across genotypes (genotype: *F*_(1,22)_ = 0.994, *p *=* *0.33; two-way ANOVA with repeated measures; Fig. 3*B*) indicating that mice from both genotypes express the same exploratory interest for the objects. 20 min after the exposition phase, both objects were moved closer to each other and mice ability to discriminate this metric change was evaluated. During the test phase, an increase in the exploration ratio (exploration duration during the test phase compared with the last 5 min of the exposition phase) is interpreted as the ability of a mouse to detect the novel spatial configuration of the objects. As shown in Figure 3*C*, as opposed to the wild types which displayed a significant increase in exploration ratio (###*p *<* *0.0001, *t *=* *8.914, df* *=* *11, index vs 50%, one-sample *t* test), *Opa1*+/− mice explored both objects equally (*p *=* *0.1806, *t *=* *1.430, df* *=* *11, index vs 50%, one-sample *t* test) and displayed a significantly lower exploration ratio than *Opa1*+/+ mice (**p =* *0.0013, *t *=* *3.675, df* *=* *22, unpaired *t* test). Thus, OPA1 haploinsufficiency results in failure to detect changes in the distance between two objects.

Overall, our data demonstrate that OPA1 deficiency leads to memory impairment in hippocampal-dependent tasks (spatial navigation in the Barnes maze, object location and pattern separation) while sparing memory in hippocampal-independent tasks (object recognition, cued Barnes maze). This phenotype reveals an important role for OPA1 in hippocampal plasticity. The hippocampal-dependent spatial tasks used here are vulnerable to adult neurogenesis deficits (Raber et al., 2004; Clelland et al., 2009; Goodman et al., 2010), which led us to ask whether stimulation of adult hippocampal neurogenesis and/or the mitochondrial system could reduce or abolish the observed cognitive defects. In this attempt, we tested the behavioral consequences of physical exercise and then the pharmacological inhibition of mitochondrial fission on memory deficits in *Opa1*+/− mice.

### Voluntary exercise corrects spatial memory deficits in *Opa1*+/− mice

**Figure 4.**
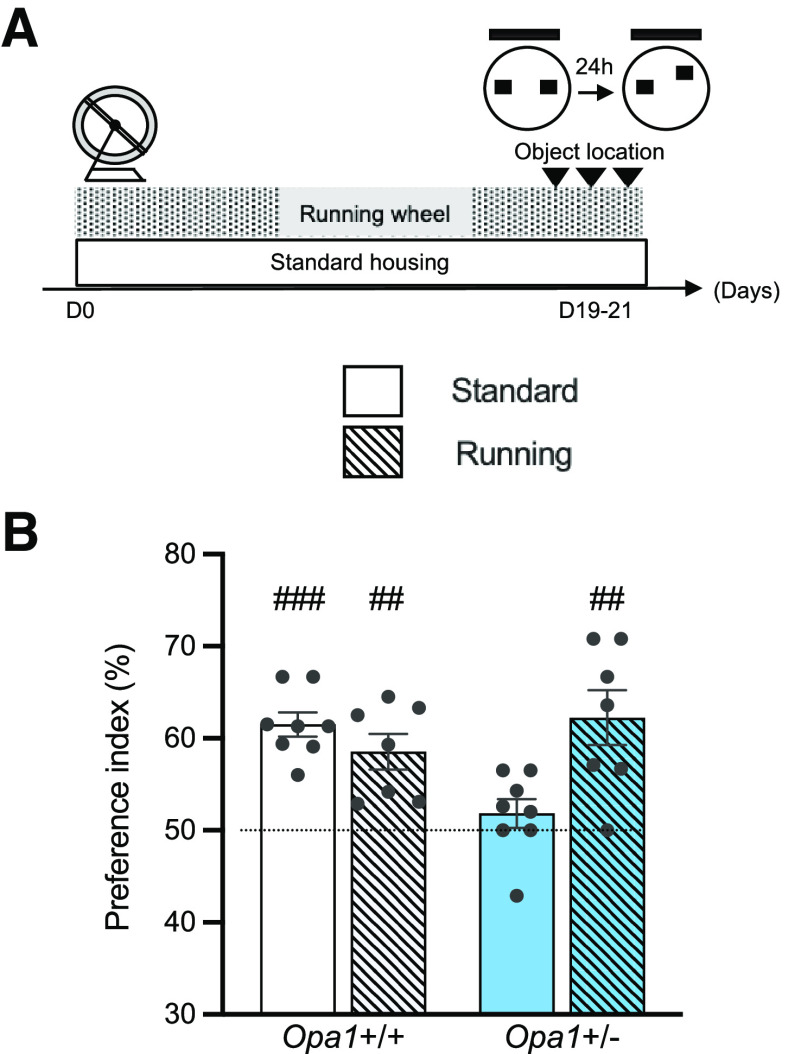
Voluntary running corrects hippocampal-dependent memory deficits in *Opa1*+/− mice. ***A***, Timeline of the protocol used to test the effect of running. ***B***, Preference indexes for the displaced object for both genotypes in standard and running conditions (*Opa1*+/+ standard, 61.50 ± 1.30%, *n *=* *8, ###*p *<* *0.0001; *Opa1*+/+ running, 58.54 ± 1.91%, *n *=* *7, ##*p *<* *0.01; *Opa1*+/− standard, 51.85 ± 1.56%; *n *=* *8, *p *>* *0.05; *Opa1*+/− run, 62.24 ± 2.99%, *n *=* *7, ##*p *<* *0.01: index vs 50%, one-sample *t* test). Dotted line indicates equal exploration of the two objects. See Extended Data Figure 4-1, voluntary running increased the number of DCX+ cells in *Opa1*+/+ and *Opa1*+/− mice and rescued mitochondrial content in *Opa1*+/−.

First, we investigated the consequences of voluntary exercise, known to enhance adult neurogenesis (Van Praag et al., 1999), on spatial memory deficits in *Opa1*+/− mice. Independent groups of mice from both genotypes were given access to running wheels or remained in standard conditions for three weeks before being tested in the object location task (Fig. 4*A*). During the exploration phase, all mice spent the same amount of time exploring each object (data not shown), indicating similar interest of the mice for the objects. During the test phase, *Opa1*+/− mice housed under standard conditions showed no preference for the displaced object (*p *=* *0.275, *t *=* *1.184, df* *=* *7; index vs 50%, one-sample *t* test), revealing a spatial memory impairment, in contrast to their *Opa1*+/+ counterparts (*t *=* *8.849, df* *=* *7, ###*p *<* *0.0001, index vs 50%, one-sample *t* test; Fig. 4*B*). However, when submitted to a three-week period of voluntary running, *Opa1*+/− mice preferentially explored the displaced object (##*p *=* *0.0064, *t *=* *4.096, df* *=* *6, index vs 50%, one-sample *t* test) as their wild-type counterparts (##*p *=* *0,0043, *t *=* *4.452, df* *=* *6, index vs 50%, one-sample *t* test; Fig. 4*B*), revealing their ability to distinguish the new location from the familiar one. Thus, voluntary exercise corrects the spatial memory deficit of OPA1-deficient mice.

10.1523/ENEURO.0073-23.2023.f4-1 Extended Data Figure 4-1 Voluntary running increased the number of DCX+ cells in *Opa1*+/+ and *Opa1*+/− mice and rescued mitochondrial content in *Opa1*+/−. Mice of eight/nine months of age were housed in standard conditions (standard) or submitted to a three-week period of voluntary running (running). ***A***, Number of DCX+ cells; *Opa1*+/+: standard, *n* = 5, 554.4 ± 54.10; running, *n* = 7, 1187 ± 194.4; *Opa1*+/−: standard, *n* = 5, 572.4 ± 91.66; running, *n* = 7, 1134 ± 138.6, *p <* *0.05, unpaired *t* test. ***B***, Running increases the mean volume of somatic mitochondria per 100 μm^3^ of GFP+ cell volume by 33%: *Opa1*+/− mice (5.072 ± 0.475; *n* = 5) compared to *Opa1*+/− mice housed under standard (3.76 ± 0.328; *n* = 7) conditions (*p <* *0.05, *U* = 158, one-tailed Mann–Whitney test). Download Figure 4-1, EPS file. Voluntary running increased the number of DCX+ cells in *Opa1*+/+ and *Opa1*+/− mice and rescued mitochondrial content in *Opa1*+/−. Mice of eight to nine months of age were housed in standard conditions (standard) or submitted to a three-week period of voluntary running (running) (***A***) Number of DCX+ cells ; *Opa1*+/+: standard, *n* = 5, 554.4 ± 54.10; running, *n* = 7, 1187 ± 194.4, **p = 0.0065, unpaired *t* test; *Opa1*+/−: standard, *n* = 5, 572.4 ± 91.66; running, *n* = 7, 1134 ± 138.6, *p < 0.05, unpaired *t* test. (***B***) Running increases the mean volume of somatic mitochondria per 100μm3 of GFP+ cell volume by 33%: *Opa1*+/− mice (5.072 ± 0.475; *n* = 5) compared to *Opa1*+/− mice housed under standard (3.76 ± 0.328; *n* = 7) conditions; *p < 0.05, *U* = 158, one-tailed Mann-Whitney test).

Moreover, as known for wild-type mice, this protocol resulted in an increase in the number of DCX1+ neurons per DG in wild-type (554.4 ± 54.1 vs 1187 ± 149.4; **p = 0.0065, *t* = 3.424, df = 10, unpaired *t* test) as well as in mutant mice (572.4 ± 91.66 vs 1134 ± 138.6; *p = 0.0118 *t* = 3.074, df = 10, unpaired *t* test; Extended Data Fig. 4-1*A*). These data validate our voluntary exercise protocol in eight- to nine-month-old mice and further suggest that rescue of adult neurogenesis defects (occurring in the critical period of new-born neurons) by exercise may be a mechanism involved in abolishing the behavioral deficits observed in *Opa1*+/− mice subjected to running. Since wild-type mice remember the location of the object whether or not they were submitted to voluntary running, an increase in neurogenesis can hardly lead to a higher performance, because of a ceiling effect in this task.

In addition, exercise also results in a 33% increase in somatic mitochondrial content of adult-born neurons (per 100 μm^3^ of GFP+ cell volume) in *Opa1*+/− mice (3.32 ± 0.33) compared with *Opa1*+/− mice housed under standard (3.32 ± 0.33) conditions (*p =* *0.0298, *U *=* *158, one-tailed Mann–Whitney test; Extended Data Fig. 4-1*B*). Given the known powerful rescuing effects of exercise on adult hippocampal neurogenesis deficits as well as on mitochondrial biomass reduction in mice (Steib et al., 2014), our findings suggest that voluntary running may act on the same parameters to correct spatial memory deficits in *Opa1*+/− mice.

### Pharmacological stimulation of mitochondria restores spatial memory in *Opa1*+/− mice

**Figure 5.**
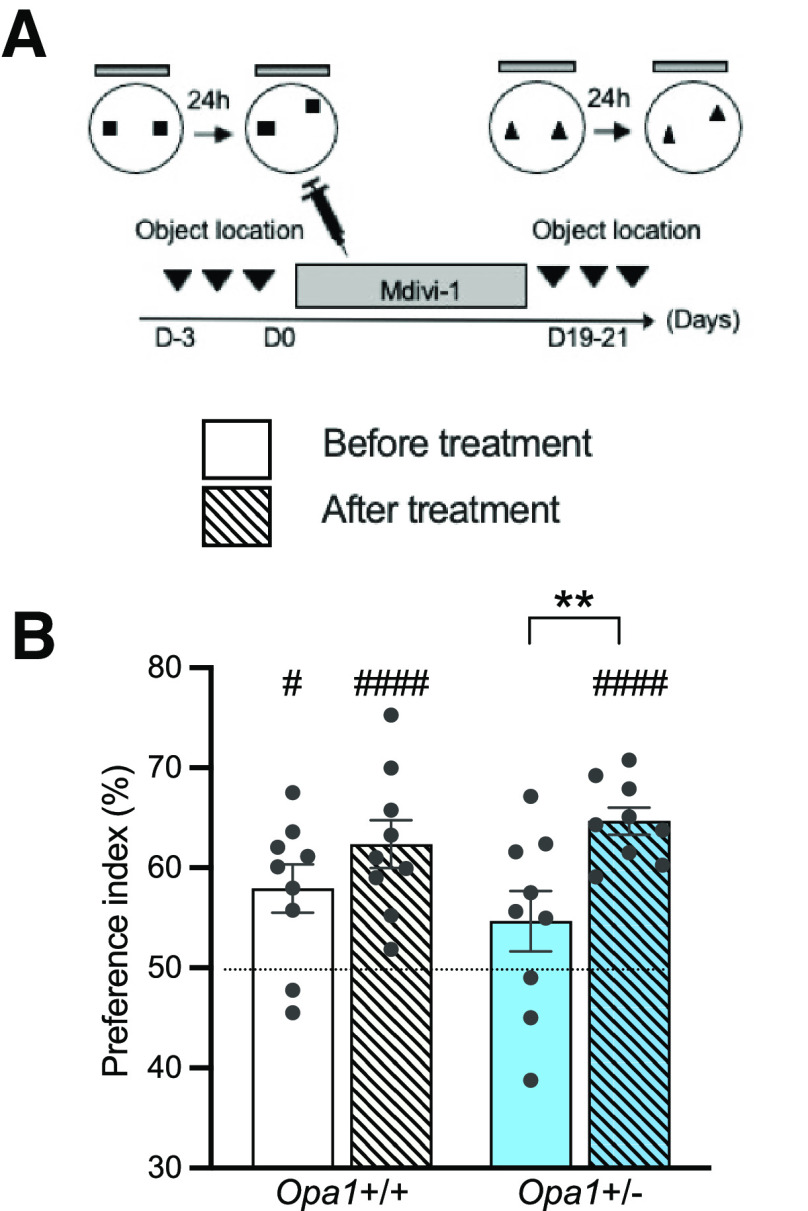
Mdivi-1 treatment corrects hippocampal-dependent memory deficits in *Opa1*+/− mice. ***A***, Timeline of the protocol used to test the effect of Mdivi-1 treatment. ***B***, Preference indexes for the displaced object for both genotypes in standard and running conditions *Opa1*+/+ before treatment: 57.94 ± 2.41%, *n *=* *9, #*p *<* *0.05, index versus 50%, one-sample *t* test; after treatment: 62.37 ± 2.41%, *n *=* *9, ###*p *<* *0.0001, index versus 50%, one-sample *t* test; *Opa1*+/− before treatment: 54.67 ± 3.01%, *n *=* *9, *p *=* *0.158; index versus 50%, one-sample *t* test; after treatment: 64.67 ± 1.35%, *n *=* *9, ###*p *<* *0.0001, one-sample *t* test. Dotted line indicates equal exploration of the two objects.

To target mitochondria, we used Mdivi-1, an inhibitor of the mitochondrial fission protein DRP1 (Cassidy-Stone et al., 2008; Rosdah et al., 2016), known to compensate for the lack of fusion associated with OPA1-deficiency. This pharmacological compound was proven efficient in animal models of neurodegenerative diseases caused by mitochondrial dysfunction (Reddy, 2014). Thus, *Opa1*+/− and *Opa1*+/+ mice received either Mdivi-1 or vehicle injections (three times a week for three weeks) and their memory was tested in the object location paradigm before and after treatment (Fig. 5*A*). During the exploration phase, all mice spent the same amount of time exploring each object (data not shown), indicating that Mdivi-1 administration had no impact on the mice’s interest in the objects. As expected, before treatment, *Opa1*+/− mice showed no preference for the displaced object (*p *=* *0.158, *t *=* *1.554, df* *=* *8, index vs 50%, one-sample *t* test), in contrast to *Opa1*+/+ littermates (#*p *=* *0.011, *t *=* *3.298, df* *=* *8, index vs 50%, one-sample *t* test; Fig. 5*B*). Remarkably, after treatment with Mdivi-1, *Opa1*+/− mice significantly explored the displaced object (###*p *<* *0.0001, *t *=* *10.90, df* *=* *8, index vs 50%, one-sample *t* test), indicating that they were now able to discriminate the new location from the familiar one, like their *Opa1*+/+ counterparts (*t *=* *5.138, df* *=* *8, ###*p *=* *0.0009, index vs 50%, one-sample *t* test; Fig. 5*B*). Consequently, treatment with Mdivi-1 restored spatial memory of *Opa1*+/− mice.

Thus, both physical exercise and pharmacological treatment targeting mitochondrial dynamics correct hippocampal-dependent memory deficits in *Opa1*+/− mice. As performance in these tasks is highly dependent on adult neurogenesis (Clelland et al., 2009; Sahay et al., 2011), our data suggest that recruitment of adult-generated neurons into hippocampal neural networks supporting this type of memory is particularly vulnerable to mitochondrial dysfunction and can be restored by acting on mitochondria.

## Discussion

Our data show for the first time that maturing adult-born hippocampal neurons are particularly vulnerable to the alteration of mitochondrial dynamics induced by a reduction of the mitochondrial inner membrane protein OPA1 in mice. In *Opa1*+/− mice, the reduced number of new neurons and their limited connectivity within the hippocampal network may contribute to cognitive impairments. Here, we provide correlative evidence that an indirect action on the mitochondrial content of hippocampal adult-born neurons corrects memory deficits in OPA1-happloinsufficient mice.

Stem cell proliferation and differentiation depend on controlled events including regulation of mitochondrial dynamics (Bressan and Saghatelyan, 2020; Giacomello et al., 2020; Chakrabarty and Chandel, 2021). Beyond the well-known shift from glycolysis to oxidative phosphorylation, pioneer gain-of-function and loss-of-function studies targeting key actors of mitochondrial functions and dynamics showed that distinct metabolic states are critical for each developmental step of neurons born during adulthood in the hippocampus, particularly during early lineage progression (Beckervordersandforth et al., 2017; Khacho et al., 2017; Wani et al., 2022). In line with these data, we find that OPA1 haploinsufficiency leads to impairments in the survival, connectivity, and mitochondrial content of adult-born granule neurons. However, the restricted OPA1 content in the *Opa1*+/− mouse model impacts neither proliferation nor neuronal fate of neural stem cells in the hippocampus. Nevertheless, newborn cells display reduced survival, suggesting a lack of pro-survival events during this period. Neurons generated during adulthood go through a “critical period” of maturation wherein dendritic arborization and spinogenesis take place, two processes requiring proper mitochondrial functions and dynamics (Khacho and Slack, 2018; Coelho et al., 2022). In line with this, OPA1 deficiency is associated with defects in spine density as well as mitochondrial content of adult-born granule neurons. Specifically, mitochondria are smaller, and along dendrites may thus be less able, during their critical period, to support spinogenesis and the high neuronal activity sustaining memory processes.

Among spine subtypes, stubby are considered immature, whereas thin and mushroom ones are known to be associated with active synapses (Nimchinsky et al., 2002). All these subtypes show reduced density in *Opa1*+/− mice, further documenting the intricate interplay between mitochondrial dynamics and synaptic connectivity in the mouse brain. In maturing primary neurons, mitochondrial defects because of OPA1 down-regulation lead to impaired spinogenesis (Li et al., 2004; Bertholet et al., 2013). Reciprocally, synaptic activity was recently shown to regulate the number of spine-associated mitochondria (Rangaraju et al., 2019; Seager et al., 2020). In the adult hippocampus, maturing new neurons display highly plastic properties that favor their functional integration (Jessberger and Kempermann, 2003). Our findings showing that OPA1 reduction impinges on the critical period of maturation of adult-born neurons thus suggest that synaptic activity might be limited in the dentate gyrus of *Opa1*+/− mice.

Consistent with the decrease in spine density of adult-born neurons observed in *Opa1*+/− mice, we provide the first evidence of spatial memory defects fully installed in these mice at middle age (eight to nine months). Only lower performances in the pattern separation task were detected in four-month-old animals (data not shown) although bearing alterations in adult neurogenesis, suggesting that cumulative alterations in adult neurogenesis contribute to changes in the cognitive abilities of *Opa1*+/− mice at older ages.

The here reported defects are indeed attributable to cognitive deficits since the visual ability of *Opa1*+/− mice is maintained at that stage, as we have observed using conventional vision-based tests (including the Barnes maze and object recognition; Pinto and Enroth-Cugell, 2000). Like most DOA patients, DOA mouse models display late-onset visual deficits because of optic nerve degeneration (Lenaers et al., 2021). Likewise, using another DOA mouse model than the one we used, a recent study reported cognitive impairments at older age (over 14 months), when visual impairments are described, thus precluding reliable determination of the cognitive profile of these mice (Bevan et al., 2020). Here, we demonstrate that eight- to nine-month-old OPA1-deficient animals display normal locomotor activity, motivation, and anxiety level. They also show intact performances in cued (visual nonspatial) memory test and in hippocampal-independent recognition test at middle age.

In the dentate gyrus of the hippocampus, adult-born neurons within their critical period of maturation and integration are repeatedly shown to play a crucial role in memory processes related to spatial navigation and pattern separation (Goodman et al., 2010; Sahay et al., 2011). Hence, we associate the cognitive impairments in *Opa1*+/− mice evidenced here in object location and metric-spatial pattern separation tasks with defects in these maturing neurons. However, the impairment might not be strictly cell autonomous, since the integration of maturating adult-born neurons in the surrounding tissue relies on various intrinsic and extrinsic parameters, including adaptation to the balance between excitatory and inhibitory synaptic inputs (Toni and Schinder, 2015). Individually and collectively, all these parameters might be susceptible to OPA1 haploinsufficiency. Nevertheless, to the best of our knowledge, no mitochondrial morphologic defect was reported in other brain regions in these mice and there is no other cognitive consequence at middle age than spatial memory defects. In brain extracts, OPA1 haploinsufficiency is known to be associated to mitochondrial dysfunctions that build up with age, starting at 10 months of age mainly as a pro-oxidative stress, sensitizing neurons to further challenges or insults. However, during embryonic development, the halved levels of OPA1 seem to have no detectable consequences that would impinge on brain maturation nor on behavior at middle-age, suggesting compensatory mechanisms. Thus, altogether our results strongly suggest that OPA1 haploinsufficiency mainly impacts adult-born neurons during their critical period when they sustain spatial memory.

Neighboring astrocytes are also likely to play a role in hippocampal functions and astrocytic mitochondria were recently involved in the regulation of adult neurogenesis (Richetin et al., 2020), calling for further investigation.

Although numerous parameters are involved in the diverse physiological consequences of voluntary exercise, free access to running wheels is not only known to enhance adult neurogenesis (Van Praag et al., 1999) but also to potentiate the reciprocal interactions between mitochondria and maturation of adult-born neurons (Steib et al., 2014). Thus, although further work would be necessary to address the underpinning mechanisms, the rescuing effects that we observe on spatial memory and on new neurons mitochondrial biomass, in *Opa1*+/− mice further suggest a role for mitochondria in these defects. Exercise may improve OPA1-related mitochondrial defects through a combination of several effects, including increased mitochondrial biogenesis (through PGC-1α activation), control quality as well as anti-oxidant mechanisms. All together, these events may compensate the low amount, in standard condition, of “healthy” mitochondria available at the vicinity of the spine necks where mitochondria sustain connectivity, and thus may favor survival of new-born neurons during their critical period.

More specifically, mitochondrial dynamics appears crucial, as administration of Mdivi-1, a pharmacological inhibitor of the mitochondrial fission protein DRP1 (Cassidy-Stone et al., 2008), counteracted spatial memory deficits in *Opa1*+/− mice. Mdivi-1 was chosen as the reference drug used to target mitochondrial dynamics through fission, in the absence of reliable alternative. Whether or not the *in vivo* effects of Mdivi-1 treatment are direct or indirect, or related to other mitochondrial parameters than DRP1 inhibition, as suggested by some recent reports (Aishwarya et al., 2020), remains to be investigated. Nevertheless, besides from being repeatedly proven to be neuroprotective, *in vitro* (Cui et al., 2010; Zeng et al., 2022) as well as *in vivo* in an Alzheimer’s disease context (Wang et al., 2017), Mdivi-1 treatment reduced the loss of newborn neurons in a mouse model of traumatic brain injury (Fischer et al., 2016).

Here, in *Opa1+/−* mice, inhibiting the mitochondrial fission protein DRP1 is thought to compensate for the lack of fusion events induced by the loss of OPA1, and restore the mitochondrial fusion/fission balance, as demonstrated in primary neurons (Lassus et al., 2016). In granule adult-born neurons, which are particularly sensitive to OPA1 reduction, it is possible that treatment with Mdivi-1 could provide sufficient functional mitochondria that could reach immature spine necks all along the dendrites and support spine maturation.

Spines maturation, synaptic connectivity and mitochondrial availability at the spine necks appear interdependent. In the context of OPA1 haploinsufficiency, although exercise might be more prone to enhance mitochondrial biogenesis while Mdivi-1 is supposed to target mitochondrial fission, we might hypothesize that both exercise and Mdivi-1 treatment will in the end converge in rescuing mitochondrial availability for dendritic spines in new-born neurons during the critical period where they sustain spatial memory.

Altogether, these data point to mitochondrial parameters and particularly their dynamics, as a putative therapeutic target.

Overall, demonstrating the reversibility of the consequences of OPA1 deficiency on spatial memory highlights the key involvement of mitochondrial dynamics in the unique plasticity of adult-born neurons. Mitochondria-based defects could therefore be revealed at an early stage of the DOA pathology by spatial memory tests, calling for further investigation in patients with DOA. Finally, the beneficial effects of exercise and of pharmacological targeting of mitochondrial dynamics on adult neurogenesis-dependent cognitive processes open new therapeutic possibilities for neurodegenerative diseases.

## Synthesis

Reviewing Editor: Erica Glasper, The Ohio State University Decisions are customarily a result of the Reviewing Editor and the peer reviewers coming together and discussing their recommendations until a consensus is reached. When revisions are invited, a fact-based synthesis statement explaining their decision and outlining what is needed to prepare a revision will be listed below. The following reviewer(s) agreed to reveal their identity: Linda Dansereau, Gandhervin Kesavamoorthy.

Overview: In this study, the authors investigated the role of mitochondria in spatial memory via a mouse model deficient in a mitochondrial fusion protein OPA1. Using histochemical and behavioral assays, the authors report alterations in adult hippocampal neurogenesis that was correlated with impairments in spatial memory. These observed deficits in spatial memory were reversed with voluntary exercise and treatment with a mitochondrial division inhibitor (Mdivi-1). The pharmacological and running data are intriguing and are of a quality that is typical of what is published in <i>eNeuro</i>. While the authors have been highly responsive to reviewer critiques, the manuscript would be better received if the authors addressed the remaining concerns below.

Remaining Concerns

1. The authors significantly rebut the rejection of the initial manuscript, providing scientific rationale as counterpoints to reviewer comments. This rationale was not incorporated into the revised manuscript. The manuscript should be revised to include the rationale and counterarguments, where appropriate scientific justification is needed.

2. All figures should be included in the main text of the manuscript. Figure 3 was not available to view in this revised manuscript.

## Author Response

Major Concerns

1. eNeuro values scientific studies that are based on scientific excellence and rigor. The use of only male mice suggests that sex as a biological variable was not considered. Please explain why only male mice were used in these experiments.

We agree that ideally these experiments should have been conducted on males and females, in order to address the issue of sex influence. This is something we are now doing systematically in our experiments, in accordance with the European recommendations.

In this study, we did use males in order to be able to compare with previous results.

2. The authors confirm dysregulated mitochondria function, in the OPA1 deficient model and demonstrated fewer adult-born surviving neurons and then provided a foundation for impaired synaptic connectivity (Line 383 - 384). However, the authors then shifted the reader’s attention to attempt to connect this with metabolic process. Since dysregulated mitochondria is part of metabolic perturbation, this disrupts the transition without a proper introduction. Please consider an introduction on how OPA1 interconnects with the metabolic process and its relationship to adult neurogenesis within the introduction (it may be suitable to do this around line 62). Repeating this citation in line 385 will improve the transition. This is like the described format in line 569.

We thank the reviewer for underlying this point. Since we did not investigate the metabolic process, we changed the sentence “Because mitochondria support the metabolic needs of adult neurogenic processes, we next examined the mitochondrial content of adult-generated neurons in Opa1+/- mice” to “Because mitochondrial functions are critical for adult neurogenic processes...” (line 392).

3. Please provide clarity in the number of mice used in the behavioral and histological experiments. N sizes per group for each outcome measure should be provided.

We apologize. N values were missing for 1 sub-group (Fig 5) and corrected line 935 (Opa1+/- before treatment : 54.67% {plus minus} 3.01, n=9, p=0.158; index vs 50%, one sample t-test;), and for DCX+ cells, corrected in legend to

Extended Figure 4-1.

4. It is unclear why the authors only targeted one aspect of mitochondrial dynamics with pharmacological treatment.

Please include findings from other pharmacological treatments

We chose Mdivi-1 as the reference drug used to target mitochondrial dynamics. There is no real alternative, all putative molecules were not thoroughly characterized.

5. The authors state that filopodia-type spines did not differ by genotype, but these data were not shown. Please provide these data.

The following data were added line 387 : filopodia: Opa1+/+: 0.35 {plus minus} 0.06 vs Opa1+/-: 0.2 {plus minus} 0.05 spines/10μm; p=0.0633, U=435.5

6. In line 58, the authors mention the quantification of mitochondria protein for OPA1 +/-, but the information generated for the current manuscript was not convincing. While homozygosity is lethal, haploinsufficiency is the main mechanism in heterozygous mutants, leading to a reduction in the OPA1 protein levels to about 50% (Lenaers et al., 2021). DOA models leading to a reduction of OPA1 level to about 50% are properly described in the cited review. We added a reference to western blot analyses done with this mouse line (Millet et al., 2016) in the introduction (line 58 and in the M&M section line 89). Supplementary Figure 1-1 describes mitochondria in the GFP reporter model. It is unclear if this image is (a) from OPA1 +/- or wildtype (b) and the comparison to littermates is missing. This image is from wild-type mouse brain and meant as an illustration of images quality with respects to the quantification method. Comparison with images from mutant mice was added in Extended Figure 1.

7. Please report mitochondrial distribution in both distal and proximal dendrites.

This piece of data was not included because the number of distal dendrites available for quantification was too low to reach statistical interest, since with age, the total number of new-born neurons decreases.

8. Please clarify the age of the mice that the DCX+ cell analysis after running was performed, indicating whether it was in 4-month-old mice or in 8-9-month-old mice matching those in the behavioral studies.

We apologize for this confusion. This was further specified. Quantification of DCX+ cells in 8-9 month old mice is only a mean to validate the running protocol, which -as expected- increases this parameter both in wt and in +/- mice. Therefore, it was moved to Extended Figure 4-1. 2 The text now reads (line 528) : These data validate our voluntary exercise protocol in 8-9 month old mice and further suggest that rescue of adult neurogenesis defects by exercise may be a mechanism involved in abolishing the behavioral deficits observed in Opa1+/- mice subjected to running.

9. It is unclear from the present study if running improved the survivability of the new neurons and increased neurogenesis. Performing this analysis may assist in interpreting the increase in neurogenesis in both genotypes, but no increase in memory ability for the wild-type mice. Running is long known to increase cell proliferation and neurogenesis in the adult mouse dentate gyrus (Van Praag et al., 1999). Here, we are reporting that voluntary exercise restores spatial memory that was impaired for Opa1+/- mice in the object location test. However, since wild-type mice already achieved a successful score statistically above 50%, an increase in neurogenesis can’t induce a higher performance (mice do not explore only the displaced object).

Therefore, “no increase in memory ability for the wild-type mice” can be expected.

10. There are many concerns related to Figure 4 and the related text (Line 517 - 525):

a. The recovered memory deficits demonstrated by the preference index in Figure 4B and the number of DCX+ cells in Figure 4C go in parallel with improved synaptic morphologies as shown in Figures 1D, 1E, and 1F as well as the mitochondrial scores displayed in Figures 1G, 1H, and 1I (all needed to establish a strong relationship with exercise). To decide whether reversed cognitive scores are at least dependent on exercise in OPA1 deficient mice, missing data must be included.

As the authors may know, there are many factors that influence cognitive outcomes in exercise-based models, ranging from secreted exokines to parenchymal derived neurotrophic factors. Given this, a difference relating to OPA1 should be clearly defined.

We agree with the reviewer that exercise induces many different pathways but our point here is to report that exercise does allow spatial memory recovery in OPA1-deficient mice, in which this capacity was specifically impaired. We did not address further OPA1-dependent mechanisms, which would require further investigations that were not planned here.

b. Running should be considered as a co-variate, as it is a highly variable behavior in mice. Please report whether running in each cage was measured or controlled for in some manner. This will assist with concerns related to differences in running in this design (inter- and intra-mice variation related to running time and intensity?). This could indeed be informative but running was not monitored in each cage per individual mouse.

c. Please discuss how exercise improved OPA1 related mitochondria defects.

Exercise may improve OPA1-related mitochondrial defects through a combination of several effects including increases in mitochondrial biogenesis (PGC-1alpha activation), control quality as well as anti-oxidant (redox state) mechanisms, ... therefore compensating for the low amount, in standard condition, of “healthy” mitochondria available at the vicinity of the spine necks where mitochondria sustain connectivity, and thus survival of new-born neurons during their critical period.

d. Please discuss how exercise supports the increase in DCX+ cells in OPA1 +/- mice to levels like wildtype mice (Figure 4C).

Density of DCX+-cells is similar in Opa1+/- and Opa1+/+ and is increased in both genotypes to reach similar levels after exercise. This suggest that several exercise-related factors are at play, which are not strictly dependent on OPA1 quantity. The problem in Opa1+/- DG is not the quantity of immature neurons (2-week old) but new neurons survival and connectivity during their critical period (3-4 week-old), both of which being supposed to benefit from exercise-induced effects.

e. Please discuss whether exercise-induced changes observed in this manuscript are mitochondria dependent or mitochondria independent?

We agree with the reviewer that exercise induces many different pathways, mitochondria dependent or mitochondria independent, but our point here is to report that exercise does allow such memory recovery in OPA1- deficient mice. We indeed did not address OPA1-dependent mechanisms, which would require further investigations that were not planned here.

This matter was discussed, line 663: Although numerous parameters are involved in the diverse physiological consequences of voluntary exercise, free access to running wheels is not only known to enhance adult neurogenesis (Van Praag et al., 1999) but also to potentiate the reciprocal interactions between mitochondria and maturation of adult-born neurons (Steib et al., 2014).

f. Line 523 states that the data not shown, however, this is related to major concern in point 5 that is one of the key figures for OPA1 +/- group. 3

In addition, exercise also results in a 33% increase in somatic mitochondrial content of adult-born neurons (per 100μm3 of GFP+ cell volume) in Opa1+/- mice (3.32 {plus minus} 0.33) compared to Opa1+/- mice housed under standard (3.32 {plus minus} 0.33) conditions (not shown; *p=0.0298, U=158, one-tailed Mann-Whitney test).

We added these data to Extended Figure 4-1.

g. Overall, more data are needed to claim that exercise directly or indirectly increases the likelihood of observing beneficial effects on memory by reversing deficits related to mitochondria in OPA1 haploinsufficient mice.

Here, the point is that we are demonstrating for the first time such a beneficial effect of exercise on spatial memory in a mitochondria-based condition due to OPA1 deficiency (haploinsufficiency). We did not claim to provide such answers, which are anyhow very unlikely to even be absolutely proven since, by definition, exercise is multifactorial.

11. There are many concerns related to Figure 5 and the related text:

a. Please explain how exercise independent factors (e.g., pharmacological treatment) improved memory. Mdivi-1, a pharmacological inhibitor of the mitochondrial fission protein DRP1 is supposed to compensate for the lack of fusion events induced by the loss of OPA1. This is a way to restore mitochondrial dynamics (fusion/fission balance), as demonstrated in primary neurons (Lassus et al., 2016). Delivered IP as a pharmacological treatment, this compound is expected to have various direct and indirect effects.

However, in DG new-born neurons that are particularly sensitive to OPA1 reduction, fission inhibition by Mdivi-1 might result in a high enough quantity of functional mitochondria that will reach immature spine necks all along the dendrites and sustain spine maturation and proper connectivity of new-born neurons during their critical period.

b. Please describe the changes associated with pharmacological treatment for DCX+ cells, neuron morphology and mitochondria as shown in Figure 1. This was not addressed in this study.

c. Please discuss the similarity/mode of action/mechanism for improved memory effects in both exercise and pharmacological treatment, including whether they act differently to improve memory or whether both interventions can correct memory deficits in the OPA1 model converging on same target.

We can speculate further, but overall, the main converging hypothesis is a mitochondrial mean to sustain spines maturation and connectivity of new-born neurons during their critical period. Mdivi-1 is supposed to target mitochondrial dynamics while exercise might be more prone to enhance mitochondrial biogenesis (PGC-1alpha).

12. The authors attempted to address the relationship between OPA1 and the memory deficits; however, the balance between mitochondria and neuronal component failed to reach a firm conclusion by the end of the manuscript. Given this the title should be reconsidered.

The title exactly reports an association : Mitochondrial OPA1 deficiency is associated to reversible defects in spatial memory related to adult neurogenesis in mice

If we had demonstrated a causal relationship, we would have written so and would have send our work for publication in a journal requiring mechanistic studies.

Although the reference was cited, if the authors are describing the OPA1Q285STOP model in Line 604, they should discuss the resource availability for this model.

We fail to understand what is meant here and why availability of this model, properly referenced, would be a concern in our study.

Minor Concerns

1. Please include the NeuN staining methods even though the results were only discussed and not shown.

This was added in the M&M section, line 149 and 180-185.

2. Please discuss why some Opa1+/- data points for the number of mushroom spines were higher than any wild-type data points. Please discuss why these mice may have been so different from the other mice.

Those three data points (over 34) for Opa1+/- that are higher than any wild-type data point for mushroom spines may reflect a higher heterogeneity for this spine category.

3. Please line edit the manuscript for typographical errors (e.g., missing words, wrong word choice).

Please provide a list of such errors that we failed to notice other than those detailed in other items.

4. Please revise figure legends with the goal of removing errant information that may have remained from previous versions of this manuscript.

For example: a. Figure 1’s legend should include a brief description of the schematic and the reference “(G,L)” should be changed to “(G-L)”.

Apologies, mistake corrected.

Figure 1’s legend was completed with : (A) Schematic illustrates the maturation steps of adult-born neurons (in days) within the dentate gyrus of the hippocampus in mouse, and specifically their critical period (21 to 28 days).

b. Please clarify the legend in Figure 2. Specifically, lines 879-880 states “In F and G, dotted lines indicate equal exploration of the two objects.” This does not appear relevant to this figure.

Apologies, mistake corrected.

c. Please clarify the legend in Figure. Line 904 refers to “standard and running conditions”, however, the figure refers to the pharmacological treatment study. 4

Apologies, mistake corrected.

d. Please edit the legend in Supplementary Figure 1-1. Clarify whether the figure is depicting a soma of a single neuron within the dentate gyrus or if there are multiple neurons depicted.

Given the scale bar, the figure depicts a single neuron.

5. Please complete the reference for Beckervordersandforth R, et al. (2017) by listing all authors.

We have corrected this mistake and thank the reviewer for such an extremely accurate proof-editing
